# Poly(Ionic Liquids) and Ionogels for Electrochromic Devices: Material Design and Additive Manufacturing Strategies

**DOI:** 10.3390/gels12030245

**Published:** 2026-03-13

**Authors:** Tatiana G. Statsenko, Ekaterina P. Baturina, Anna A. Nikitina, Sofia M. Morozova

**Affiliations:** 1 Moscow Center for Advanced Studies, Kulakova St. 20, 123592 Moscow, Russia; 2 Center of Soft Matter and Physics of Fluids, Bauman Moscow State Technical University, 2nd Baumaskaya St. 5/1, 105005 Moscow, Russia; 3 Infochemistry Scientific Center, ITMO University, Lomonosova St. 9, 192001 Saint-Petersburg, Russia

**Keywords:** materials design strategy, electrochromic materials, electrochromic devices, poly(ionic liquids), ionogels, 3D printing

## Abstract

Escalating requirements for smart energy management are driving advances in functional electrochromic devices (ECDs), which are pivotal for the regulation of light, heat, and reduction in energy consumption in buildings, transportation, and smart devices. However, the commercialization of ECDs is hindered by com plex designs, high fabrication costs, and slow switching speeds. Additive manufacturing (AM, 3D-printing) emerges as a promising approach to overcome these limitations, as it enables the creation of complex structures, enhances design flexibility, and can reduce production costs. For such printed devices, materials combining poly(ionic liquids) (PILs) with ionogels—an emerging and promising class of materials known for their high ionic conductivity, stability, and tunable properties—are particularly suitable for integration with 3D printing. Comparing previous reviews that address PILs, ionogels, or AM modalities in isolation, this work uniquely combines the structure–property–processing relationships specific to the synergistic integration of these fields. Current work highlights recent progress in PIL/ionogel-based ECDs and distills specific design guidelines for optimizing ink rheology, balancing ionic conductivity with mechanical integrity, and selecting appropriate printing modalities. These insights provide a roadmap for overcoming current fabrication challenges and scaling up next-generation smart devices.

## 1. Introduction

**Figure 1:**
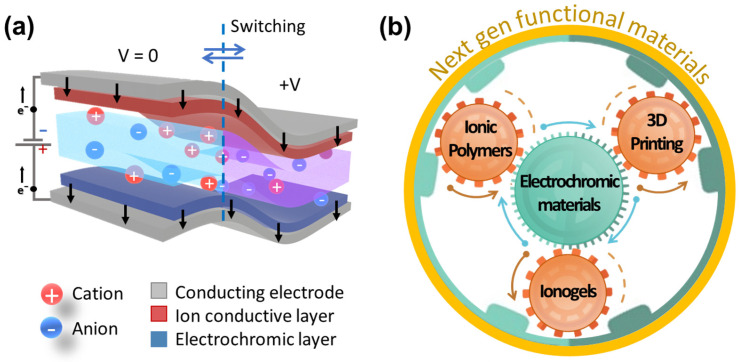
Next-generation ECDs: (**a**) typical device architecture employing a polymer electrolyte, where a polymer matrix (depicted as gray chains) serves as the host for mobile ions; (**b**) conceptual roadmap illustrating the relationship between ionic polymers, ionogels, and AM (e.g., DIW and DLP), showing that this collaboration is yielding new, next-generation functional EC materials.

Electrochromism—the reversible and controllable alteration of a material’s optical characteristics through applied voltage—serves as the fundamental operating principle for electrochromic devices (ECDs, Figure 1a) [1,2]. This functionality relies on distinct color-changing mechanisms in various material classes, such as the reversible Li^+^ ion intercalation in inorganic metal oxides (e.g., tungsten trioxide, WO_3_, and vanadium pentoxide, V_2_O_5_) [3] the redox-induced optical transitions in conducting polymers (e.g., polyaniline [4] PEDOT:PSS) [5], and the electrochemical properties of organic molecules (e.g., viologens) [6]. Figure 1a represents the architecture of a modern ECD, where the electrolyte acts as a critical bridge, governing the ion flux that drives the optical transition. The most prominent application lies in energy-efficient smart windows for architectural and automotive glazing, which can intelligently regulate solar heat gain and glare, thereby reducing energy consumption. Beyond this, ECDs are integral to the development of low-power, bistable reflective displays for electronic paper, signage, and wearable electronics, and have been integrated with supercapacitors to create multifunctional devices that visually signal their state of charge [7,8,9]. The scope of their application continues to expand into niche yet critical areas such as adaptive military camouflage and advanced sensing platforms [10,11].

The operational core of a conventional ECD comprises several essential components: an electrochromic (EC) layer that undergoes reversible optical changes, transparent conductive electrodes that enable uniform charge distribution, and an electrolyte that serves as the ion-conducting medium to facilitate charge balance during redox processes. While early ECD development predominantly utilized rigid inorganic materials, recent advances have shifted toward flexible polymer-based systems compatible with emerging flexible electronics.

Among these components, the electrolyte plays a critical role in determining device performance. An ideal electrolyte must exhibit high ionic conductivity to ensure rapid switching kinetics, exceptional electrochemical stability to withstand prolonged operation under applied voltages, and high optical transparency in its passive state [12]. Traditional high-conductivity liquid electrolytes have drawbacks such as leakage, volatility, and potential toxicity which compromise the durability and safety of the device [13,14].

In this context, ionic polymers and ionogels have emerged as promising candidates for next-generation electrolytes. Ionogels are quasi-solid-state materials in which an ionic liquid, as a dispersed phase, is confined within a three-dimensional polymer or inorganic matrix [15]. By analogy, with hydrogels and organogels—where water or an organic solvent serves as the liquid phase, respectively—ionogels synergistically combine the mechanical robustness of a solid with the high ionic conductivity of a liquid electrolyte. This structure effectively immobilizes the liquid component, yielding non-volatile and mechanically robust electrolytes [16,17]. In turn, poly(ionic liquid)s (PILs) where ions are covalently bonded to the polymer backbone offer the excellent processability of polymers combined with the inherent ionic conductivity and stability of the parent ionic liquids (ILs) [18]. The combination of PILs and ionogels can enhance component compatibility and further increase the overall ionic conductivity of the electrolyte. The molecular tunability of these ionic systems allows for the precise engineering of materials with tailored properties. This includes, in particular, the development of single multifunctional layers where all active components—EC, ion-conducting, and electrode materials—are integrated into a “single multifunctional layer” [19].

A transformative leap in this field is the integration of these materials with AM, which allows for direct, single-step fabrication of custom devices (Figure 1b) [20]. As illustrated in the roadmap in Figure 1b, the convergence of PIL/ionogel chemistry with modalities like DIW and DLP enables the transition toward monolithic optoelectronics. This strategy both simplifies production and unlocks novel form factors [21] such as flexible and conformal electronics [22,23].

The gap between PIL/ionogel chemistry and AM processing makes it possible to overcome the economic and design limitations of current technologies. While numerous excellent reviews have independently addressed advances in EC materials [24,25,26,27,28] the development of ionic conductors like PILs and ionogels [17,29,30] or the application of AM for electronic devices [31,32] these domains are typically treated in isolation. Consequently, a comprehensive analysis dedicated to the synergistic integration of these three pillars—materials, electrolytes, and fabrication—for creating next-generation ECDs is currently lacking. This review aims to fill this critical gap by analyzing the synergistic integration of these three pillars—materials, electrolytes, and fabrication. The review begins by providing a concise overview of the fundamental principles and historical development of ECDs. This is followed by an in-depth analysis of advanced ionic materials, focusing on the relationship between their structure and properties. Subsequently, the review explores the transformative role of various AM techniques in fabricating architectured ECDs. Finally, the review concludes by critically assessing the current challenges and offering a perspective on future research directions at the intersection of these fields.

## 2. Evolution and Commercialization of ECD: From Monofunctional Systems to Multifunctional Platforms

**Figure 2:**
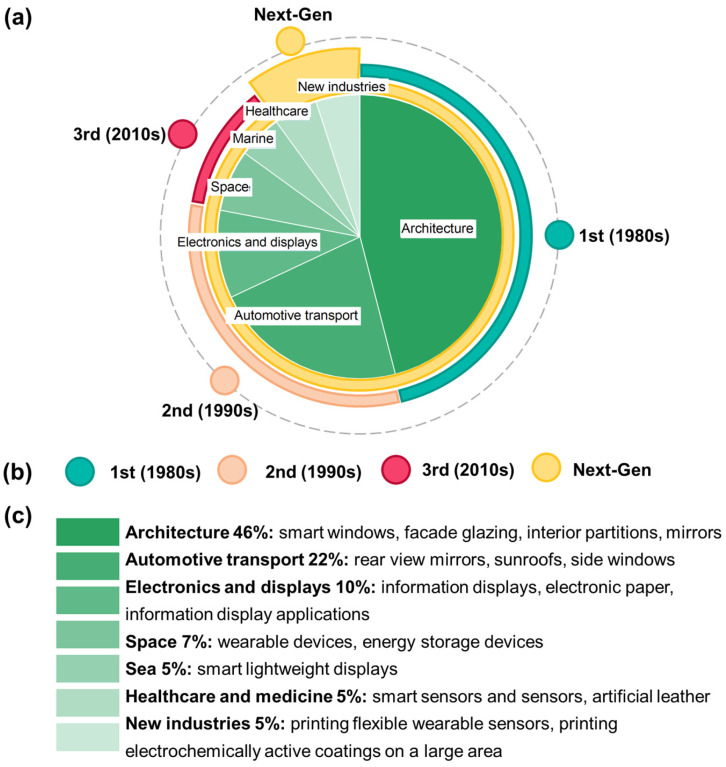
Evolution and commercialization of ECDs: (**a**,**b**) the historical trajectory is the transition from monofunctional components to specialized multifunctional materials; (**a**,**c**) industries in economic sectors using EC materials and devices.

Conventional ECD have been historically based on a multilayer architecture, typically comprising inorganic oxide films (e.g., WO_3_) as the active layers and a liquid electrolyte. These first-generation systems, while effective, were inherently monofunctional and limited by mechanical fragility, potential electrolyte leakage, and constraints to simple planar geometries. The historical trajectory of ECs thus reveals an irreversible shift from these discrete components toward purpose-built, multifunctional platforms (Table 1, Figure 2a,b). The advent of materials with programmable ionic, mechanical, and optical properties, such as PILs and ionogels, combined with the topological freedom of AM, now enables the fabrication of geometrically complex ECDs unattainable by conventional methods.

This chapter traces this technological evolution by first discussing the shift to multifunctional materials (Section 2.1), then exploring the advanced fabrication enabled by AM (Section 2.2), and concluding with an analysis of the commercialization and market outlook (Section 2.3).

### 2.1. The Multifunctional Materials Evolution

The electrochromism phenomenon was first identified by Platt in 1961 [33] In 1969, Deb demonstrated this effect using a thin film of amorphous WO_3_ (1 µm thick) and conducted pioneering research that marked the advent of the “first generation” of ECDs [34]. These devices focused on singular optical modulation had several disadvantages: fragile inorganic layers (e.g., NiO [35], V_2_O_5_ [36]), prone-to-leakage liquid electrolytes, and manufacturing processes (e.g., sputtering, chemical vapor deposition [37,38]) inappropriate for complex geometries. Concurrently, early research also explored organic electrochromes, most notably viologens [6] which demonstrated reversible color changes in solution and laid the groundwork for future molecularly engineered materials [39]. The subsequent “second generation” in the 1990s saw the widespread adoption of conductive polymers such as polyaniline (PANI) [4,40] and poly(3,4-ethylenedioxythiophene):poly(styrene-4-sulfonate) (PEDOT:PSS) [5,41]. These materials are characterized by enhanced mechanical flexibility but constrained by interfacial instability between the electrolyte and the device. By the 2000s, hybrid organic–inorganic systems [42,43] were developed in an attempt to merge conductivity with manufacturability. However, significant improvements in cycling stability were not achieved; it remained below 10^3^ cycles.

The 2010s marked a paradigm shift with the rise of the “third generation” of materials, initiated by the introduction of ILs [44] as non-volatile, safe alternatives to conventional liquid electrolytes. This innovation quickly evolved into the development of PILs [45,46] and ionogels [47,48], which solidified the electrolyte and unlocked true multifunctionality with the following advantages:

- *Ion Transport and Charge Balancing:* PIL networks, engineered with tailored counterions (e.g., TFSI^−^, BF_4_^−^), achieve ionic conductivities exceeding 10^−2^ S·cm^−1^ by optimizing segmental chain dynamics [49].
- *Mechanical Stabilization:* Self-healing ionogels (DLP-printed acrylate/PIL hybrids) reduce delamination through dynamic hydrogen bonding [50].
- *Intrinsic Electrochromic Activity:* Viologen-functionalized PILs integrate coloration and ion conduction into a single macromolecular framework [51].

The evolution summarized in Table 1 illustrates a clear trajectory from discrete, monofunctional components toward highly integrated, multifunctional materials.

**Table 1:** Multifunctional evolution of EC materials.

Generation (Time Period)	Materials	Key Advances	Key Limitation	References
**1st** (1980s)	Inorganic oxide (WO_3_, NiO); Liquid electrolytes; Viologens	High optical modulation	Mechanical fragility; Electrolyte leakage risk	[39,52]
**2nd** (1990s)	Conducting polymers (PANI, PEDOT:PSS)	Mechanical flexibility; Solution processability	Poor cycling stability (<500 cycles); Interfacial instability	[53]
**3rd** (2010s)	ILs; PILs; Ionogels	Multifunctionality (ion conduction & coloration); Non-volatility & improved safety; Tunable properties	Scalability barriers	[44,47]
**Next-Gen** (2020s+)	3D-printable inks based on PILs/ionogels	Integrated manufacturing; Architectural freedom (3D geometries); Rapid prototyping & customization	Interlayer compatibility challenges; Material–process co-design complexity	[54]

The evolution summarized in Table 1 illustrates a clear trajectory from discrete, monofunctional components toward highly integrated, multifunctional materials. However, unlocking the full potential of these third-generation systems requires a parallel revolution in manufacturing. Conventional fabrication methods are inadequate for exploiting the unique properties and design freedom offered by PILs and ionogels. This challenge is now being addressed by integrating these advanced materials with AM, or 3D printing. AM enables the co-design of material, device architecture, and function, paving the way for the next generation of EC systems with unprecedented complexity and performance [55,56,57]. This synergy will be the focus of the subsequent section.

### 2.2. AM for Advanced ECDs

Conventional manufacturing of ECDs, reliant on sequential, often analog, layer-by-layer deposition and assembly, has historically limited the integration of multifunctional components into monolithic structures. The transition from analog layer-by-layer assembly to digital AM workflows enables architectural freedom. Among the diverse suite of AM technologies, several have proven particularly suitable for fabricating next-generation ECDs [58,59,60]:

- *Direct Ink Writing (DIW):* Exploits the shear-thinning behavior and thixotropy recoveryof ionogels, to align functional fillers, enhancing conductivity by orders of magnitude. This technique facilitates the structuring of ionogels, leading to the alignment of tubular fibers and a remarkable 100-fold enhancement in ionic conductivity [61].
- *Digital Light Processing (DLP):* Relies on rapid sol-gel transitions triggered by UV-cross-linking, achieving response times below 100 ms. By utilizing a photocurable composition based on polymerizable ionic monomer, ionogels with high sensitivity, rapid response times (64.2 ms), and exceptional durability over 1000 cycles have been produced [62].
- *Multi-component Inks with DIW:* The combined use of multi-component inks with DIW promises to develop highly elastic and long-lasting alternating current electroluminescent (ACEL) devices with inconsistent structures, paving the way for future production of ionotronics [63].
- *Photolithography:* Electrochemical microdevices based on ionogels, fabricated from thiol-acrylate precursors in the presence of ionic liquid, exhibit a low Young’s modulus of 0.23 MPa and high ionic conductivity up to 2.4 × 10^−6^ S/cm with 75 wt% incorporated ionic liquid [64].

Despite this rapid progress, significant challenges remain in translating these proofs-of-concept into reliable technologies. A primary hurdle lies in materials engineering—specifically, the formulation of functional inks with precisely tailored rheological profiles. This involves engineering properties like shear-thinning behavior (thixotropy) [65,66] for extrusion-based methods like DIW and controlling photopolymerization kinetics for vat polymerization techniques like DLP [67,68]. Another critical challenge is ensuring robust interfacial adhesion and compatibility between dissimilar printed materials. Finally, the long-term electrochemical stability of the active materials, such as preventing the oxidative degradation of PILs at operating voltages above 4.0 V, remains a key concern for device longevity. Overcoming these multifaceted challenges is crucial for advancing printed ECDs from laboratory prototypes to commercially viable products.

### 2.3. Market Dynamics

Driven by the global transition towards a sustainable economy, the ability of EC materials to dynamically regulate energy flows has propelled them from laboratory curiosities to commercially significant products, fostering a market projected to exceed $20 billion by 2030 with a compound annual growth rate (CAGR) of over 8% (Figure 2) [69,70].

While the overall EC materials segment is already substantial—projected to grow from $901.5 million in 2024 to $2 billion by 2034 [71]—the economic case for next-generation devices is significantly strengthened by AM. The integration of ionic polymers and ionogels using 3D printing is not merely a technological leap but a strategic economic advantage. By enabling the monolithic fabrication of complex architectures with integrated electrolytes and electrodes in a single, digital process, AM directly addresses key manufacturing costs.

This approach is reported to reduce production costs by 30–40% [72] and material consumption by 60% [29,73] compared to conventional methods. By lowering the barrier to entry and enabling scalable production, AM is a critical enabler for the market’s expansion into new applications. This growth is evident as leading players actively invest in deploying these technologies across real-world sectors:

- *Automotive Industry:* Mercedes-Benz utilizes EC “Magic Sky Control” sunroofs (SPD technology), which reduce interior temperatures by 10 °C and reserve energy [74]. Renault integrated “Solarbay” sunroofs with PDLC technology [75,76] in 2023. Gentex Corporation (automotive EC mirrors) saw smart glass revenue in the transport segment reach $3.5 billion in 2023 [77,78].
- *Building Sector:* EC windows reduce building energy consumption by 20% (data from View Inc. and Nabr, 2022) [79,80]. Integration with Internet of Things (IoT) systems [81] for lighting and temperature control is exemplified by View Inc.’s projects at Phoenix Airport [82,83,84]. AGC Inc. and NSG Group reported a 15% increase in profits from EC architectural solutions between 2023 and 2024 [84].

In summary, this chapter has traced the evolution of ECD from rigid, monofunctional systems to integrated, multifunctional platforms. Advances in materials science, particularly the development of PILs and ionogels, are intrinsically linked to the paradigm shift in manufacturing brought about by AM. This synergy not only unlocks unprecedented device performance and design freedom but also provides the economic impetus necessary for widespread commercial adoption, setting the stage for the next generation of smart materials.

## 3. Poly(Ionic Liquid)s in ECD: Materials Engineering for Multifunctional Applications

Poly(ionic liquid)s (PILs) represent a cornerstone in the development of next-generation, solid-state ECD. Their unique molecular architecture allows for the precise tuning of electrochemical, mechanical, and optical properties, making them ideal candidates for multifunctional applications. This chapter provides a comprehensive overview of PILs in the context of ECDs, charting a course from fundamental material design to advanced device integration. Current section begins by exploring the core structure–property relationships that govern PIL performance, focusing on polymer backbones and ionic moieties (Section 3.1). Critical ion transport and interfacial dynamics mechanisms that determine device performance are discussed (Section 3.2). Building on this foundation, advanced molecular engineering strategies that enable novel functionalities, such as stimuli-responsiveness, are explored (Section 3.3). Finally, advanced integration approaches where PILs are synergistically combined with other EC layers and host polymers to achieve enhanced performance and durability (Section 3.4).

### 3.1. Architectural Principles of PILs

**Figure 3:**
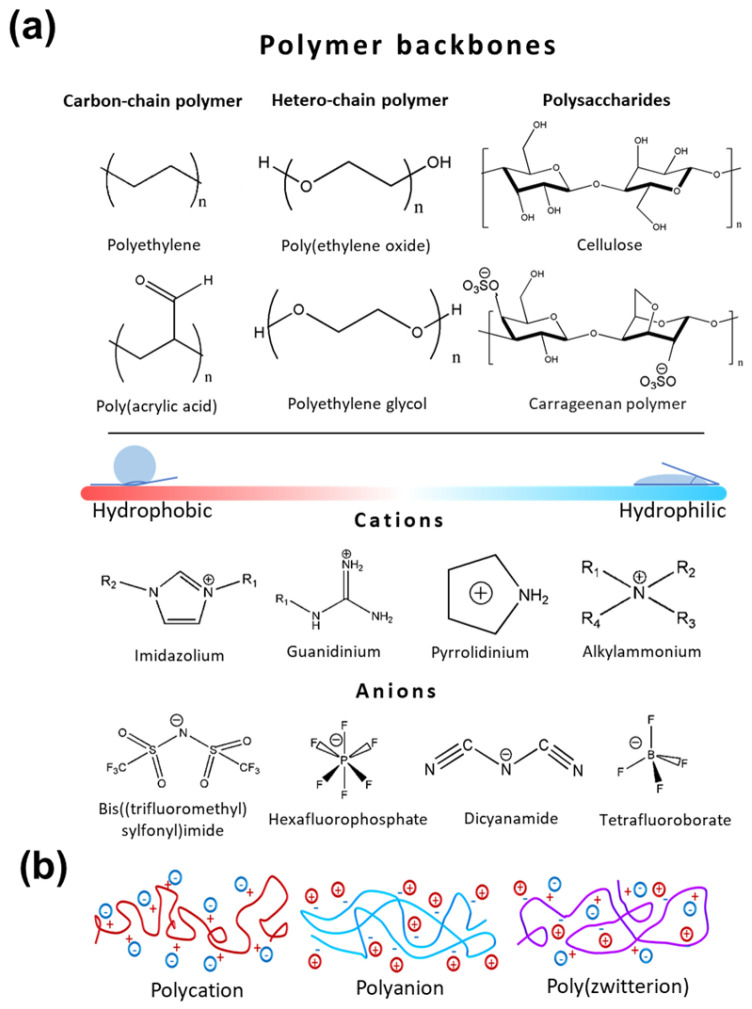
Structure and classification of poly(ionic) liquids (PILs): (**a**) the commonly used polymer backbones, cations, and anions for polycation-type ILs; (**b**) PILs based on the location of the charge on the polymer backbone.

Poly(ionic liquid)s (PILs) represent a class of polyelectrolytes with a polymer chain and ionic liquid (IL) fragments in each of the repeating units (Figure 3a) [49,85,86]. ILs themselves are a unique class of molten salts, defined as having melting points below 100 °C, which consist of asymmetric structure ions that prevent crystallization, resulting in a transition to the liquid state under normal conditions [87]. By covalently linking these ionic groups to a polymer, PILs synergistically combine the inherent processability [88] and mechanical integrity of polymers with the high ionic conductivity, thermal stability, and negligible volatility of ILs. While PILs have been explored for several decades in various electrochemical applications, their recent integration with advanced fabrication techniques like 3D printing has revitalized the field, making them exceptionally promising materials for next-generation ECDs [29,89]. Depending on the charge of polymer backbone, PILs are divided into polycations, polyanions, and polyzwitterions (Figure 3b).

As illustrated in Figure 3, the design of a PIL involves the strategic selection of two primary components: the polymer backbone and the ionic fragments. Commonly used polycationic PILs feature polymer chains such as polyethylene (PE), polymethyl methacrylate (PMMA), or heteroatom-containing backbones like poly(ethylene oxide) (PEO) that provide tunable film-forming capacity, mechanical strength, and optical transparency [69,90,91,92]. These macromolecular backbones often exhibit low glass transition temperatures (T_g_) and higher ionic conductivities than those bearing heteroatomic substituents. To create multifunctional materials, electroactive chains such as polythiophene [93] or polypyrrole [94] can be integrated to introduce intrinsic EC activity and enhance charge transport [95]. Unlike traditional salt-in-polymer electrolytes, PILs eliminate ion leaching due to the covalent tethering of the charge carrier.

The ionic moieties consist of cationic groups paired with mobile anions (imidazolium, pyridinium, ammonium, pyrrolidinium or BF_4_^−^, PF_6_^−^, and more commonly, [N(SO_2_CF_3_)_2_]^−^ (TFSI)) could be covalently grafted onto these polymer backbones [91,96,97]. Polycationic PILs, particularly those based on imidazolium, are preferred for ECDs due to their wide electrochemical stability window (>3.0 V) and low glass transition temperature (T_g_), which facilitates rapid ion hopping [98,99] PILs have dual roles in ECDs: as ion-conducting electrolytes and as electroactive layers. Their high ionic conductivity (up to 10^−3^–10^−2^ S·cm^−1^) [100,101], low volatility, and thermal stability (>200 °C) directly improve device switching speed (<1 s), cycling stability (>10^4^ cycles) [89]. For example, PILs incorporating redox-active moieties such as viologen or aniline derivatives reveal intrinsic electrochromism, allowing for simplified device architectures [102]. Moreover, their compatibility with solution-processing techniques facilitates the fabrication of flexible and large-area ECDs [103,104].

For optimal performance in ECDs, side reactions during redox cycling should be prevented by providing the electrochemical neutrality and stability of the constituent ions. Polycationic PILs are demonstrating the highest electrochemical stability compared to polyanionic and polyzwitterionic PILs (due to sulfonate (–SO_3_^−^) or carboxylate (–COO^−^) functional groups). Therefore, polycationic PILs are more commonly used in contemporary ECD design [105,106]. Key strategies to enhance ionic conductivity in these systems include designing monomers with the highest chain flexibility, copolymerization with amorphous polymers to suppress crystallinity, and modification of functional groups with electronegative fragments [107].

### 3.2. Electrolyte Interfaces and Ion Transport Mechanisms

The performance of PIL-based ECDs is critically dependent on the dynamics at electrolyte–electrode interfaces and the efficiency of ion transport through the bulk material. This integration addresses several challenges in conventional EC systems, such as high interfacial resistance and ion trapping at the electrolyte–electrode boundary, while enabling novel functionalities [108,109,110].

**Figure 4:**
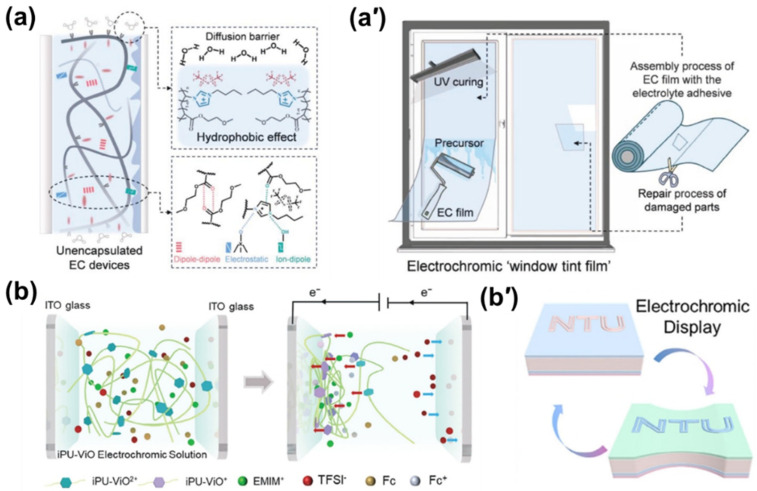
Poly(ionic liquid)s in ECDs: (**a**) schematic illustration of structural properties for unencapsulated EC devices based on P(IL-MEA) electrolyte, (**a′**) assembly, installation, and repair process of unencapsulated, removable EC “window tint film” [111]; (**b**) schematic diagram of the EC process of the iPU-assembled EC device switching from the initial 0 to −0.9 V; (**b′**) schematic microstructures of sensing ion skin [112].

A promising example involves viologen- and ferrocene-based ILs functionalized with polyethylene glycol side chains and paired with bis(trifluoromethanesulfonyl)imide ([TFSI]^−^) anions. This molecular design provides exceptional compatibility with common IL electrolytes such as [EMIm][NTf_2_], which allows for increased solubility of the components. These systems exhibit impressive performance, achieving 79.4% optical modulation and retaining 96.2% of their initial contrast after 1000 cycles across a wide operational temperature range (−30 to 70 °C). Ultra-thin (10 μm) films maintain rapid switching kinetics (3.7 s coloring, 13.3 s bleaching), demonstrating the power of precise molecular engineering [113].

Building upon these foundational designs, significant research has focused on enhancing device durability and stability. Innovations in this area include leveraging stable C–F bonds in fluorinated anions to result in minimal water uptake and wide electrochemical stability windows. This strategy facilitates the fabrication of robust, unencapsulated EC window films at a potentially low cost (~$110/m^2^), thereby eliminating the need for stringent, inert-atmosphere manufacturing environments while achieving exceptional cycling stability (Figure 4a,a′) [111].

Further advancements are coming from controlling the fundamental mechanism of ion transport. For example, anion-selective ionic conductors (ASICs), such as polyaniline, have been engineered to optimize the performance of specific EC polymers. By achieving a high anion transference number (0.75) and substantial ionic conductivity (2.41 × 10^−3^ S/cm), these conductors enhance switching efficiency, enabling novel applications such as adaptive camouflage fabrics that can dynamically shift color from earthy-yellow to dark-green [114]. Driving toward sustainability has encouraged the development of biopolymer-based electrolytes, such as carrageenan composites, a natural polysaccharide, with the IL [EMIM][SCN]. The ionic conductivity of these systems can be tuned over seven orders of magnitude (from 2.3 × 10^−11^ to 4.6 × 10^−4^ S/cm), with an optimal IL concentration of 15 wt% yielding fast switching (6 s oxidation, 8 s reduction) and near-perfect optical modulation (99%) at very low operating voltages (0.3 to −0.9 V) [115].

### 3.3. Advanced Functionalities and Stimuli-Response

Beyond fundamental structural design, significant research has focused on tailoring PILs to introduce advanced functions crucial for next-generation devices.

A primary focus is the systematic enhancement of ionic conductivity. Molecular engineering approaches, such as the quaternization of N-substituted imidazoles with various side-chains, have proven effective. For instance, PILs based on TFSI anions with alkyl side chains demonstrate lower T_g_ values and higher conductivities (4.7 × 10^−6^ S/cm at 25 °C) compared to those with heteroatomic substituents. This conductivity can be further increased to 1.0 × 10^−5^ and 4.5 × 10^−4^ S/cm at 25 and 70 °C, respectively, when the TFSI anion is replaced with BF(CN)_3_^−^. Such materials show excellent electrochemical stability (>3.2 V vs. Ag^+^/Ag) and thermal stability (>185 °C), making them prime candidates for solid-state electrolytes [116].

Another powerful strategy to overcome the classic tradeoff between mechanical robustness and high conductivity is the development of cross-linked polymer networks. This approach involves designing mechanically robust and highly conductive solid-state electrolytes by incorporating ionic liquid-based cross-linked polymer networks. The resulting cross-linked membranes exhibit substantial mechanical strength, with a Young’s modulus of 40–50 MPa, while maintaining high ionic conductivity in the range of 4 × 10^−4^ S/cm at 60 °C [117]. Notably, the high concentration of ethylene glycol moieties into the PIL structure significantly enhances ionic conductivity and allows for the incorporation of a larger amount of the lithium salt compared to the alkyl-substituted analogs. This unique combination of properties positions the developed membranes as promising candidates for application in solid-state lithium batteries.

A key frontier is the development of cross-linked ionogel networks that decouple mechanical strength from conductivity. This is achieved by incorporating photochromic moieties, such as diarylethenes (DAEs), directly into the PIL structure. Upon light irradiation, the DAE unit undergoes a reversible ring-opening and -closing isomerization, which creates a localized change in charge and conformation. This allows the binding between mobile ions and the DAE-containing cations to be controlled, thus reversibly modulating the ionic conductivity of the solid film. This elegant mechanism enables spatiotemporal control over conductivity with a maximum 70% drop upon irradiation, allowing for the creation of light-writable electronic circuits and advanced photodetectors [118]. The non-invasive nature of this photomodulation provides a unique handle for future mechanistic studies and the design of next-generation PILs with improved fatigue resistance and thermal stability.

Furthermore, in the scope of a circular economy, a new generation of sustainable and environmentally friendly materials and devices has emerged. For instance, aqueous-based electrolytes containing 1-ethyl-3-methylimidazolium thiocyanate ([Emim][SCN]) have been developed. The ionic conductivity varies with IL concentration and temperature, reaching a maximum of 119 mS/cm for 20 wt% [Emim][SCN] at 80 °C. Flexible ECDs based on these electrolytes demonstrate coloration efficiency up to 1422.8 cm^2^ C^−1^, positioning such sustainable solid polymer electrolytes as excellent candidates for environmentally friendly ECDs [119].

### 3.4. Integration with Electrochromic Layers for Enhanced Performance

Synergistic integration of PILs with other functional materials is a key strategy for developing high-performance devices. This integration addresses several challenges in conventional EC systems, such as high interfacial resistance and ion trapping at the electrolyte-electrode boundary, while enabling novel functionalities.

A promising strategy involves creating hybrid or composite systems where PILs enhance the properties of conventional host polymers [120,121,122]. For instance, modifying poly(vinylidene fluoride-co-hexafluoropropylene) (PVDF-HFP) with covalently linked imidazolium (BMIM^+^) cations nearly triples the material’s ionic conductivity (13.1 mS/cm vs. 4.7 mS/cm). This enhancement directly translates to superior device performance: increased optical modulation (74% ΔT) and unprecedented long-term stability, with 98% performance retention after 20,000 cycles. Electrochemical quartz crystal microbalance (EQCM) analysis confirmed that the PIL modification effectively suppresses cation accumulation at the electrode interface, a common failure mechanism in traditional devices [123].

Another powerful approach is the direct copolymerization of ILs with electroactive polymers. For example, an imidazolium-based ionic liquid was copolymerized with 3,4-ethylenedoxythiophene (PEDOT). It is intriguing that this copolymer shows a shorter switching time of 0.37 s, 0.30 s, and 0.45 s at 1100 nm compared with PEDOT alone [124]. This confirms that the existence of an imidazolium-based ionic liquid can improve the diffusion properties and switching time of conjugated polymers, providing a potential direction for the preparation of high-performance EC materials.

The merging of these advanced materials with novel manufacturing techniques represents the frontier of ECD development. Cationic polyurethanes based on hydroxypropyl viologen (HDPV) exemplify this trend, offering a unique combination of tunable mechanical properties (strength from 7.6 to 76.6 MPa), high optical contrast (79.1%), and an intrinsic memory effect. Crucially, the rheological properties of these materials are compatible with AM techniques (Figure 4b,b**′**) [112]. The quantitative performance metrics of these PIL-based systems, including their superior cycling stability compared to traditional electrolytes, are summarized and benchmarked in Table 2 (Section 4.3).

Finally, poly(ionic liquid)-dispersed liquid crystal (PDLC) smart windows hold significant potential for energy-efficient applications. To address challenges like high driving voltages, a pre-orientation strategy via a low-voltage electric field was developed. This approach optimizes the liquid crystal molecular alignment during the phase separation process, resulting in a 61.2% reduction in threshold voltage (from 20.6 V to 8.0 V) [120]. Crucially, a programmable patterned PDLC film was successfully obtained by using conductive photospacers, enabling stepwise control of light transmission and opening avenues for intricate architectures for broader application scenarios.

In conclusion, this chapter has demonstrated that poly(ionic liquid)s are a highly versatile class of materials whose properties can be precisely engineered at the molecular level. The strategic incorporation of functional ionic polymers, whether through blending, copolymerization, or hybridization with host polymers, enables the simultaneous optimization of ionic transport, electrochemical stability, and mechanical properties in ECDs. By carefully selecting polymer backbones, ionic moieties, and leveraging strategies like in situ polymerization, it is possible to overcome long-standing challenges such as interfacial resistance and electrochemical instability. The successful design of these multifunctional materials is paramount, as they form the foundation upon which advanced fabrication techniques can build the next generation of high-performance, durable, and commercially viable ECDs.

## 4. Ionic Liquid Gels (Ionogels) as Advanced Electrolytes in ECDs

Ionogels represent a unique class of soft matter that effectively hybridizes the rapid ionic dynamics of liquid electrolytes with the structural stability of solid-state polymers. As quasi-solid-state materials, they synergistically combine the high ionic conductivity and non-volatility of ILs with the mechanical robustness of a percolating polymer network. This chapter provides a critical analysis of ionogels, focusing on strategies to enhance their mechanical resilience, interfacial adhesion (Section 4.1), and advanced compositional engineering through supramolecular chemistry and nanomaterials (Section 4.2). Finally, Section 4.3 explores their integration into multi-responsive intelligent systems.

**Figure 5:**
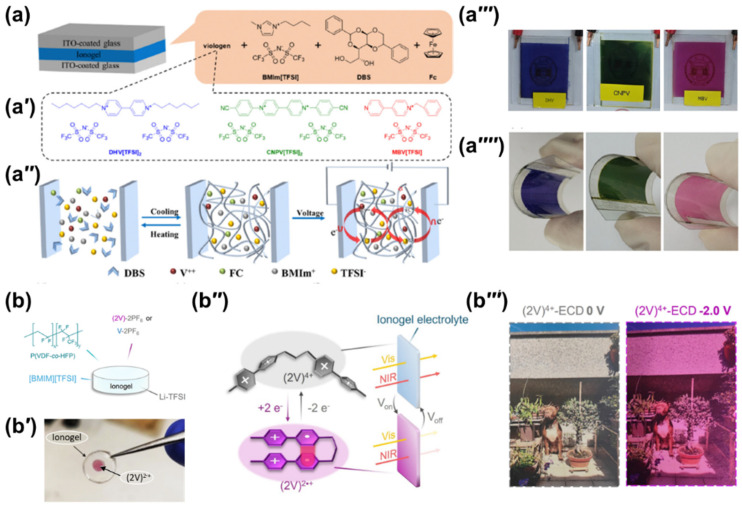
Ionogels in ECDs: (**a**) fabrication and switching mechanism of a viologen-based gel ECD (**a′**–**a′′′′**), showcasing the transition between optical states and device flexibility [30]; (**b**) NIR-responsive ECDs based on “viologen tweezers” in ionogel matrices (**b′**–**b′′′**), demonstrating enhanced solar energy modulation through intramolecular radical pairing [132].

Structurally, ionogels comprise an IL physically or chemically entrapped within a continuous three-dimensional network. This architecture immobilizes the IL, eliminating leakage and volatility issues typical of traditional systems [16,87]. While historically associated with silica matrices via sol-gel processes [15,125], the term now encompasses any gel-like composite containing an IL, whether the gelator is polymeric, colloidal, or supramolecular [15]. Substitution of the aqueous or organic phase with an IL imparts a suite of superior properties compared to conventional hydrogels and organogels, including significantly higher ionic conductivity [126], increased thermal stability, pronounced resistance to freezing [127] and intrinsic antimicrobial activity [128]. The functional role of the IL within the ionogel matrix is remarkably versatile. As illustrated in Figure 5a, the assembly of a typical viologen-based ECD involves the entrapment of redox-active species within a tailored gel matrix, where the specific molecular structure of the viologen (Figure 5a′) dictates the resulting color palette. In one configuration, the IL can be a constituent of the framework. The operational integrity of such systems relies on a well-defined working principle (Figure 5a″), where the reversible redox reactions of the gel-embedded molecules drive the optical transition under applied bias. Thus, PILs can act as the primary gelator, architecting the network structure and pore design through a combination of controlled hydrogen bonding, electrostatic forces, and host–guest interactions [129,130]. The IL functions as the liquid dispersion medium, physically entrapped within a separate polymer or inorganic scaffold [131]. In this case, the ionogel takes the majority of the IL’s advantageous physicochemical characteristics. This design flexibility allows for the rational engineering of ionogels with precisely tailored properties. For example, by selecting self-healing polymer matrices, mechanical durability can be dramatically improved, while incorporating redox-active species like viologens into the gel network can impart intrinsic EC functionality.

While the integration of ionogels into ECDs overcomes primary challenges like leakage and volatility, the transition to these quasi-solid-state systems introduces a new set of considerations, namely ensuring high mechanical robustness and robust interfacial adhesion to the electrode surfaces, which are critical for device service time and performance [133]. Furthermore, high concentrations of polymeric or inorganic gelators, required in many fabrication processes, can interfere with ion transport, resulting in reduced ionic conductivity and deficient EC behavior. For instance, performance tests conducted at specific wavelengths and voltages (Figure 5a′′′) are critical for evaluating the switching kinetics and optical contrast of these quasi-solid-state systems [30]. Consequently, contemporary research is focused on the rational design of ionogels that simultaneously exhibit superior mechanical properties, such as high flexibility during bending (Figure 5a′′′′) high ionic conductivity, and advanced functionalities [30,112,134,135].

### 4.1. Enhancing Mechanical Resilience and Interfacial Adhesion

Achieving high mechanical toughness without compromising ionic mobility is a central challenge in gel science. One successful approach is the in situ phase separation strategy during a one-pot polymerization to fabricate an ionogel electrolyte with exceptional mechanical toughness (5.33 MJ m^3^), extensibility (786%), and adhesive strength (2.18 MPa) [134]. When integrated into an ECD with poly(3,4-(2,2-dimethylpropylenedioxy)thiophene) (PProDOT-Me_2_) and Prussian blue (PB) films, this ionogel facilitated a large transmittance contrast (50%), rapid response times (1.2/1.8 s), and outstanding cycling stability, retaining over 95% of its performance after 10,000 cycles. Another strategy applies intermolecular hydrogen bonding within a poly(hydroxypropyl acrylate) (PHPA)–PMMA matrix to create a highly elastic and bistable EC ionogel [136]. Here, bistability refers to the ability to retain a colored state for an extended period (over 54 h) without continuous power consumption. This system demonstrates remarkable tensile elasticity, which streamlines the assembly of flexible devices, alongside superior EC metrics: a large transmittance change (>80%), high coloration efficiency (85.3 cm^2^·C^−1^), and robust stability.

### 4.2. Compositional Engineering and Nanostructuring Strategies

To circumvent the reduction in ionic conductivity often caused by high polymer loading, supramolecular ionogels have been developed using low-molecular-weight gelators [30]. The resulting DBS-G ionogel demonstrated self-healing properties, high optical transmittance (>86%), and ionic conductivity (3.12 mS/cm) comparable to that of the bare IL. ECDs constructed with this supramolecular gel performed comparably to liquid electrolyte systems and superior to conventional polymer-based ionogels, functioning effectively across a wide temperature range (−25 °C to 80 °C).

Advanced fabrication routes, such as electrospinning, offer another dimension of control by creating nanofibrous scaffolds. These membranes support high IL loading within interstitial pores, providing continuous pathways for accelerated ion transport. A rapid (<1 min) photopolymerization method for (PIL)-based precursors has been developed [137]. The resulting ionogels possess high transparency, elasticity, and excellent physicochemical stability, enabling the fabrication of high-performance flexible ECDs. Nanostructuring via electrospinning presents another powerful fabrication route. Flexible ECDs were fabricated by sandwiching electrorotating webs between two plastic electrodes consisting of silver nanowire/PEDOT:PSS hybrid electrodes. The fabricated device provided smooth color switching over a large active area of 10 × 10 cm^2^, with a voltage of 0 to 1.8 V, a transmittance difference of up to 39%, and a high coloration efficiency of 266.8 cm^2^/C at a wavelength of 550 nm, with transmittance maintained even after 20 bending cycles [138]. This morphology leads to significantly improved device characteristics, including accelerated switching kinetics, enhanced cycling stability, and superior coloration efficiency in both rigid and flexible device architectures. As shown in the comparative analysis in Table 2, nanostructured ionogels often exhibit a higher coloration efficiency (CE) than their bulk counterparts due to optimized ion diffusion pathways.

Further compositional engineering involves the integration of advanced porous materials, most notably Metal–Organic Frameworks (MOFs). The hybridization of zirconyl-imine MOFs with a viologen-based IL has been shown to enrich the available color states and stabilize the redox cycling process. In particular, the obtained MOFs:EV[TFSI]2 (0.5:1) ECD material exhibits a synergistic effect, with the redox cycle being stabilized for over 1100 cycles and the kinetic stability of color bleaching tested up to 1600 switches [139]. Furthermore, to address insufficient ion-doping/de-doping processes observed with monovalent cations (e.g., Li^+^), a viologen-based divalent cation gel electrolyte (VGE) was designed. This system facilitates a more comprehensive electrochemical process in PProDOT-Me_2_ films, amplifying the optical modulation range from 13.3% (with Li^+^) to 39.4% and enabling the construction of robust, low-power flexible displays [140].

Supramolecular engineering is key tool for resolving the inherent tradeoff between near-infrared (NIR) absorption and electrochemical stability in viologen-based systems. As depicted in Figure 5b, the precise loading of functional “viologen molecular tweezers” into the ionogel matrix facilitates a broader Vis–NIR response than that achieved by conventional monomeric concentrations, which typically suffer from rapid degradation [132]. This structural preorganization promotes intramolecular radical pairing, extending the absorption spectrum deep into the infrared region (Figure 5b′′), thereby significantly outperforming traditional systems in solar irradiance modulation. The macroscopic impact of this molecular design is evident in free-standing gel samples (Figure 5b′), which exhibit intense coloration upon the application of a reductive potential. For practical applications, complex sandwich-type ECDs are fabricated by a streamlined solution-coating process on ITO glass electrodes. These devices demonstrate efficient switching between fully transparent and NIR-blocking states (Figure 5b′′′) achieving a 2.4-fold higher modulation of solar heat gain compared to monomeric counterparts. Notably, the supramolecular approach allows for high optical performance at lower concentrations and reduced operating voltages, ensuring robust operational stability over at least 950 consecutive dyeing-bleaching cycles. Such results validate supramolecular design as a powerful tool for the next generation of energy-efficient smart windows.

### 4.3. Towards Multi-Responsive Intelligent Systems

A prospective frontier involves integrating multiple stimuli-responsive functionalities into a single monolithic ionogel system. The operational efficiency of the materials discussed in the previous sections can be quantitatively benchmarked to highlight the fundamental tradeoffs between mechanical integrity and electrochemical response. As summarized in Table 2, the performance metrics of representative PIL and ionogel systems reveal distinct advantages depending on their molecular architecture. For instance, while PIL-based systems exhibit unmatched cycling stability (up to 20,000 cycles) [112], supramolecular and nanofibrous ionogels typically offer higher coloration efficiencies and faster switching kinetics due to optimized ion diffusion pathways.

**Table 2:** Comparative electrochromic performance of representative PIL and ionogel-based electrolytes.

Material Class	ΔT (%)	Switching Time (s) (t_c_/t_b_)	CE (cm^2^/C)	Stability (Cycles)	Key Feature	References
**Polycationic PIL**	74.0	5.0/6.2	210	20,000	Extreme longevity	[112,123]
**Supramolecular Ionogel**	80.0	2.5/3.1	184	5000	High speed & modulation	[30]
**Nanofibrous Ionogel**	39.0	1.8/2.2	266	>500	Max. coloration efficiency	[138]
**MOF-Hybrid Ionogel**	42.0	8.0/10.0	95	1600	Redox stabilization	[139]
**Aqueous-based PIL**	68.5	6.0/8.0	1422	>1000	Ultra-high CE	[119]

ΔT: optical contrast; t_c_/t_b_: coloration/bleaching time; CE: coloration efficiency.

Building on these performance benchmarks, the prospective frontier of the field involves integrating multiple stimuli-responsive functionalities into a single monolithic system. A “single multifunctional layer” ionogel has been reported that unifies EC and thermochromic properties by incorporating a thermo-responsive polymer with electroactive species (methyl viologen and ferrocene) [141]. This device can function as a privacy window via a temperature-triggered opaque-to-transparent transition, while also exhibiting high-contrast electrochromism (ΔT = 74.9%) with excellent coloration efficiency (229.03 cm^2^·C^−1^). Similarly, a highly stretchable and conductive ionogel with UCST-type thermal sensitivity was synthesized via one-pot photopolymerization [142]. This material enables the fabrication of devices that function concurrently as temperature monitors, smart windows, and strain sensors, demonstrating the potential for ionogels to serve as the core component in truly multifunctional smart systems. The data presented in Table 2 underscores that the selection of a specific ionogel architecture is dictated by the target application—whether it requires the extreme longevity of PILs for architectural glazing or the rapid response of supramolecular networks for dynamic displays. The programmability and advanced manufacturing compatibility, such as through 3D printing, further promise the creation of complex, customized architectures for next-generation optoelectronic applications [133].

Ionogels have better electrochemical and mechanical properties than liquid electrolytes; however, their full potential remains limited by geometric constraints. It is required to transition to AM, which enables the precise fabrication of complex integrated architectures. However, translating this material-level innovation into devices with complex architectures needs a new manufacturing paradigm, provided by AM, which is the focus of the subsequent chapter.

## 5. AM as a New Paradigm for ECD Fabrication

AM, also known as 3D printing, is rapidly emerging as a transformative fabrication paradigm for ECDs. By enabling the direct, spatially controlled deposition of functional materials, this digital manufacturing approach offers a powerful alternative to conventional multi-step, cleanroom-based processes, unlocking unprecedented design freedom and facilitating the monolithic integration of multifunctional components. This chapter examines the key AM modalities that are reshaping ECD fabrication. Section 5 introduces vat photopolymerization methods (Section 5.1), extrusion-based techniques such as DIW (Section 5.2), and jetting-based systems such as inkjet printing (Section 5.3), concluding with emerging approaches such as Fused Filament Fabrication (Section 5.4). These technologies have been adapted to modern materials such as ionogels and hydrogels to create devices with new form factors and improved performance.

### 5.1. Vat Photopolymerization (SLA/DLP)

**Figure 6:**
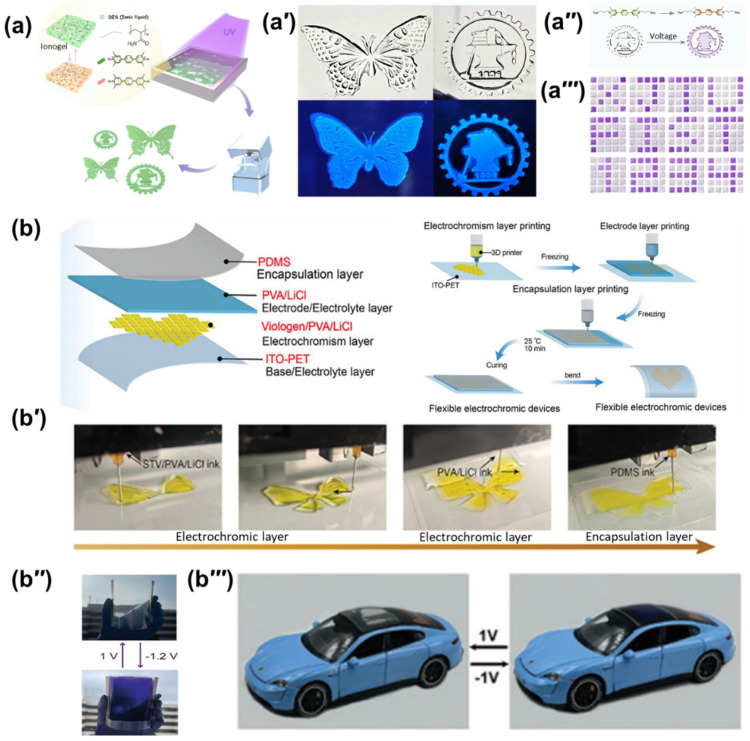
3D printing ECDs: (**a**) technical route for the one-step preparation of structured electrochromic ionogels; (**a′**–**a′′′**) snapshots of the high-transparency samples, their electrochromic switching, and alphanumeric display capabilities [54]. This figure has been published in CCS Chemistry 2025; Three-Dimensional Printable Viologen-Based Ionogel for Visible Sensing and Display is available online at 10.31635/ccschem.024.202404393; https://www.chinesechemsoc.org/doi/full/10.31635/ccschem.024.202404393, accessed on 17 February 2026; (**b**) integrated multi-material DIW process for hydrogel-based ECDs; (**b′**–**b′′′**) snapshots of the sequential layer deposition, large-area array fabrication (10 × 10 cm^2^), and integration scenarios in smart windows [55].

These techniques utilize light to selectively cure a liquid photopolymer resin, offering high resolution and geometric fidelity. They are ideally suited for fabricating ionogels from photocurable precursors. A significant breakthrough in this area addresses the challenge of photogenerated radicals, which typically inhibit polymerization. Han et al. engineered a novel phenyl viologen derivative that avoids the formation of free radicals under UV irradiation, making it fully compatible with Digital Light Processing (DLP). This innovation enabled the first DLP 3D printing of finely structured viologen-based ionogels (Figure 6a), possessing a remarkable combination of high transparency (85%) (Figure 6a′), extreme stretchability (17 times), and self-healing capabilities. The resulting materials function as multi-responsive sensors and can be patterned into complex displays, showing distinct electrochromic switching (Figure 6a′′) and alphanumeric patterns (Figure 6a′′′), demonstrating how molecular engineering can unlock new fabrication possibilities [54].

### 5.2. Direct Ink Writing (DIW)

This extrusion-based method is highly adaptable, capable of depositing a wide range of viscous inks, including polymer solutions, pastes, and ionogels [20,143,144]. The capacity for multi-material printing is particularly advantageous for creating fully integrated devices in a single process. This capability has been powerfully demonstrated by Luo et al., who utilized multi-material DIW to fabricate fully integrated, flexible hydrogel-based ECDs (FECDs) following the structural design shown in Figure 6b. By sequentially printing a viologen/polyvinyl alcohol (PVA) hydrogel ink as the active layer, a PVA/LiCl electrolyte, and a PDMS encapsulation layer (Figure 6b′), they achieved a monolithic device without complex assembly steps. The resulting FECDs exhibited exceptional performance, including high optical contrast (up to 54% at 360 nm) and remarkable cycling stability, enabling the fabrication of large-area 10 × 10 cm^2^ arrays (Figure 6b′′) for practical application in smart windows (Figure 6b′′′), showcasing the potential of DIW for streamlined manufacturing of robust, patterned devices (Figure 6b–b′′′) [55].

Transition to multi-material DIW required specific failure modes that must be managed. Unlike single-material prints, the dominant failure mechanism in these integrated devices is often interfacial delamination, driven by the mismatch in swelling ratios and elastic moduli between the hydrogel electrolyte and the adjacent electrode layers during redox cycling. Furthermore, for hydrogel-based systems, dehydration remains a primary cause of performance decay in non-encapsulated environments, necessitating the simultaneous printing of robust encapsulation layers to ensure long-term stability.

Furthermore, DIW’s versatility is exemplified in fabricating complete, multi-layered emitting devices. For instance, the precise, pixel-by-pixel deposition of specialized ZnS/PDMS composite inks has enabled the creation of flexible, transparent, and multicolor electroluminescent (EL) displays. This approach, which integrates distinct emissive layers, transparent electrodes, and encapsulation in a single process, demonstrates DIW’s capacity for monolithic device integration directly from functional inks, achieving high mechanical resilience over 20,000 bending cycles [145].

The capabilities of DIW are expanding beyond planar structures through advanced extrusion and hybrid methodologies. Coaxial extrusion, for example, facilitates the continuous fabrication of multi-material, core-shell functional fibers. This was leveraged to produce meter-long “Structro E-fibers” with a liquid metal core and a liquid crystal elastomer shell, forming the flexible foundation for a wearable device [146].

### 5.3. Inkjet Printing

Characterized by the non-contact jetting of low-viscosity inks, this method provides exceptional precision for high-resolution patterning [147]. Inkjet printing is optimized for high-resolution patterning of thin-film components, such as conductive polymer electrodes. Ma et al. utilized drop-on-demand (DoD) printing to create image-integrated smart windows with dual-mode (coloration/scattering) control at 42 DPI. A critical constraint remains the ink’s viscosity (<20 mPa·s), which necessitates significant dilution and may limit the areal ionic conductivity compared to extrusion methods [148].

While less common for depositing bulk ionogel layers, inkjet printing is exceptionally well-suited for defining thin-film components such as conductive polymer electrodes. Recent work has demonstrated its application in fabricating flexible, textile-based ECDs by printing polyaniline nanosheet inks directly onto fabric, paving the way for wearable smart textiles and displays [149].

It is important to note the inherent limitations of inkjet technology regarding material formulation. The strict requirement for low ink viscosity (typically <20 mPa·s) often conflicts with the high solid content or high ionic liquid loading needed for high-performance ionogels. High IL concentrations can disrupt stable droplet formation (satellite drops) or clog nozzles. Consequently, inkjet formulations often require significant dilution, which can result in thinner active layers and potentially lower areal ionic conductivity compared to extrusion-based methods.

### 5.4. Fused Filament Fabrication (FFF)

This widely accessible extrusion technique for thermoplastics is being explored for electrochemical applications. While direct printing of ionogels is challenging, the development of composite filaments offers a viable path forward. Research on FFF-printed electrodes for supercapacitors, using a PLA/graphite filament subsequently modified with nickel hexacyanoferrate, demonstrates the principle of embedding electrochemically active components within a printable thermoplastic matrix [150]. This strategy is directly translatable to ECDs, where PILs or ILs could be incorporated as fillers to create ionically conductive filaments. A critical limitation of FFF is the tradeoff between ionic conductivity and mechanical rigidity. ILs act as powerful plasticizers in the thermoplastic matrix. A high IL content, while desirable for conductivity, often makes the filament too soft to be effectively moved by the extruder gears without warping (filament abrasion), limiting the maximum practical conductivity of electrolytes printed using FFF.

To facilitate the selection of the most suitable manufacturing strategy, Table 3 provides a comparative overview of this AM technique. It highlights critical tradeoffs between processing parameters, such as resolution and speed, and material compatibility, guiding selecting specific technologies for specific EC applications.

As shown in Table 3, the landscape of printed ECDs is defined by inherent tradeoffs between resolution, throughput, and material compatibility. Consequently, the choice of AM technology—whether high-precision DLP and inkjet printing or the multi-material reliability of DIW—must be closely aligned with the target application. Collectively, these technologies represent a paradigm shift from traditional assembly to monolithic freeform manufacturing. However, to fully realize the potential of this technological toolkit, fundamental bottlenecks in ink formulation and interface stability must be addressed, which will be critically examined in the next section.

## 6. Artificial Intelligence and Machine Learning in ECD Development: From Predictive Modeling to Autonomous Discovery

The vast parameter space of PIL and ionogel design presents a significant challenge for trial-and-error optimization. The integration of Artificial Intelligence (AI)/Machine Learning (ML) enables a shift toward “inverse design,” where desired performance metrics dictate the material composition. Recently, the integration of AI and ML has emerged as a transformative paradigm to accelerate the development of next-generation ECDs. By leveraging computational power to extract complex correlations from multidimensional datasets, ML models enable researchers to forecast performance metrics, such as transmittance modulation and switching kinetics, while significantly reducing the experimental workload. Section 6 evaluates the impact of AI on the field, covering predictive performance modeling (Section 6.1), accelerated discovery of PIL-based ionogels (Section 6.2), autonomous self-driving laboratories (Section 6.3), and emerging applications in smart sensing and neuromorphic systems (Section 6.4), concluding with a prospective outlook on digital materials science (Section 6.5).

### 6.1. Performance Modeling and Lifetime Prediction

Accurately predicting the operational stability and performance of ECDs is most important for their commercial adoption. A pioneering study by Wu et al. [148] utilized a Long Short-Term Memory (LSTM) network to forecast the cycle life of liquid-state tungsten oxide-based ECDs. The model was trained on a high-fidelity dataset of 1000 electrochemical cycles, utilizing optical modulation (1000-cycle datasets for durability, 120-structure datasets for PILs) as the primary target metric. By analyzing the time-series degradation patterns, the LSTM achieved high predictive accuracy, identifying that D_2_O-based electrolytes extend functional life by reducing ion-trapping kinetics, a correlation extracted without exhaustive full-duration testing. 

Similarly, predictive mapping has been applied to thin-film fabrication. Faceira et al. [149] combined experimental sputtering parameters with ML to model the behavior of WO_3_ films. The researchers utilized a structured input feature set including deposition pressure, oxygen partial pressure, and discharge power. Their Random Forest and Gradient Boosting models yielded scores exceeding 0.85 for predicting color persistence and reversibility, effectively creating a “digital twin” that navigated a 300-point parameter space with minimal experimental samples. In another study, used employed seven ML regressors to optimize the electrolyte recipe in ammonium metatungstate-based systems, confirming that ML-identified concentrations achieved superior optical modulation compared to conventionally formulated samples [151].

### 6.2. Accelerating Materials Discovery: PILs and Ionogel Electrolytes

The chemical diversity of ionic materials makes them ideal candidates for ML-guided discovery. Piroozi and Kammakakam [152] addressed the challenge of ionic conductivity in imidazolium-based PILs. They created a dataset of 120 distinct PIL structures, utilizing 2D chemical descriptors and molecular weight as input features. The trained CatBoost and XGBoost ensemble models achieved a Mean Absolute Error (MAE) of less than 0.15 log units in conductivity prediction, allowing for the rapid screening of over 1000 virtual candidates.

Beyond regression models, generative AI is paving the way for de novo material design. Khajeh et al. [153] developed a transformer-based generative model to propose novel polymer electrolytes. By utilizing a closed-loop active learning framework, the model iteratively generated and evaluated polymer SMILES strings to optimize Li-ion conductivity. While initially demonstrated for battery technologies, this methodology is readily adaptable to tailoring the chemistry of PILs and ionogels for EC applications, where balancing ion mobility with mechanical integrity is critical.

The “inverse design” workflow, as exemplified by generative models [153], follows a rigorous iterative loop: (1) generation of novel polymer SMILES strings via a transformer-based architecture; (2) feature extraction of Li-ion coordination and segmental mobility; (3) performance evaluation through molecular dynamics simulations; (4) an active learning update where the most promising candidates are synthesized and fed back into the training set to refine the generative policy.

### 6.3. Autonomous Labs and Self-Driving Systems

The integration of ML with robotic automation has led to the emergence of “self-driving laboratories” (SDLs), which represent the pinnacle of digital materials science. As exemplified by the platform developed by Dahms et al. [154] for solution-processed electrochromic films, the SDL paradigm transitions research from traditional Edisonian trial-and-error toward a systematic, data-driven methodology. This approach operates as an iterative, closed-loop system designed to navigate the vast parameter space of ionogel formulations and deposition conditions without human intervention.

By coupling automated deposition with real-time characterization, such SDLs significantly boost throughput and precision. This autonomous cycle not only eliminates human bias but also ensures that the identified processing parameters are optimized for long-term device performance, effectively accelerating the discovery-to-application timeline for next-generation electrochromics.

### 6.4. Intelligent Sensing and Neuromorphic Computing

AI is also expanding the functional envelope of ECDs toward smart sensing and unconventional computing. Ranjbar et al. [155] demonstrated an EC colorimetric sensor augmented with ML classifiers for antioxidant detection. The ML algorithms decoded subtle multicolor optical patterns from polyaniline and Prussian-blue-based layers, enabling accurate identification and quantification of six different antioxidant compounds. This effect allowed the transformation of ECDs from simple light modulators into sophisticated diagnostic platforms. Furthermore, the trend toward non-destructive, real-time in situ monitoring is evidenced by the development of integrated FET-based platforms for the continuous sensing of dynamic physiological states [156].

Furthermore, the intersection of ion gels and AI extends into neuromorphic “ionotronics.” Tsuchiya et al. [157] utilized ionogel–graphene interfaces for physical reservoir computing, achieving a two-order-of-magnitude reduction in energy consumption compared to software-based ML. Such advancements highlight the broader potential of functional gels for low-power, AI-integrated hardware.

### 6.5. Future Outlook in AI–Gels Integration

As experimental datasets in polymer science continue to expand, the role of AI in ionogel research must transition from purely empirical observation toward mechanistic understanding and industrial implementation. Three key frontiers will define this evolution.

*First*, the field is moving toward Physics-Informed Machine Learning (PIML). Unlike traditional “black-box” models, PIML integrates fundamental polymer physics—such as Flory–Huggins theory for gel swelling or Nernst–Planck equations for ion transport—directly into the neural network’s loss function. This ensures that the AI-predicted behavior of a new PIL remains physically consistent, even when training data is sparse. Furthermore, the adoption of explainable AI (XAI) techniques, such as SHAP (SHapley Additive exPlanations), will be paramount in distilling “design rules” from complex models, allowing researchers to quantify how specific chemical features (e.g., cation–anion binding energy) impact macroscopic metrics like coloration efficiency and switching kinetics.

*Second*, addressing the “small data” challenge in ionogel synthesis requires the implementation of Transfer Learning and Active Learning frameworks. By leveraging models pre-trained on massive chemical databases (e.g., ChEMBL) and fine-tuning them on high-quality experimental datasets of ionogel switching cycles, researchers can achieve high predictive accuracy with significantly fewer experiments. This digital maturation is essential for realizing the 30–40% cost reduction potential of printed ECDs mentioned in the Introduction. AI-driven optimization minimizes material waste by predicting successful “ink recipes” before they are physically formulated, effectively lowering the R&D barrier for industrial scale-up.

*Third*, the ultimate frontier lies in the integration of AI with real-time 3D-printing control and long-term stability monitoring. Future autonomous systems will utilize computer vision and Bayesian-active learning to perform in situ error correction, adjusting extrusion pressures or UV intensities on-the-fly to compensate for batch-to-batch variations in ink rheology. For ECDs to succeed in automotive and architectural sectors, AI must also be used to model “real-world” degradation. This involves training models on multi-environmental stress tests (UV exposure, thermal cycling, and humidity) to predict the service life of unencapsulated ionogels. By overcoming these bottlenecks, printing throughput, material cost, and operational durability, AI-guided digital materials science will transition electrochromic technologies from niche laboratory prototypes to scalable, high-performance smart optoelectronic platforms.

## 7. Conclusions and Future Perspectives

The integration of PILs and ionogels with AM represents a transformative frontier in ECD technology. This review highlighted the combination of PILs’ molecular versatility with the high ionic conductivity of ionogels, thereby addressing the historical limitations of liquid-state ECDs, such as leakage, instability, and rigid design constraints. However, as the field moves toward commercial viability, several interrelated challenges and future directions must be considered.

The primary hurdle remains optimizing ink rheology and gelation kinetics. For extrusion-based printing (DIW), the ink must exhibit a delicate balance between shear-thinning behavior and rapid structural recovery (thixotropy) to maintain shape fidelity without compromising ionic transport. In vat photopolymerization (DLP/SLA), high concentrations of ILs often act as plasticizers, reducing cross-linking density and leading to mechanical fragility or phase separation (syneresis).

Furthermore, mechanical durability and interfacial engineering are critical for the longevity of 3D-printed ECDs. Traditional ionogels are often prone to delamination when integrated into multi-material stacks (e.g., electrode/electrolyte interfaces). Developing “toughened” ionogels through double-network structures or supramolecular interactions—including self-healing and stretchable motifs—is essential for the next generation of wearable and conformal optoelectronics.

The future of printed electrochromics lies in the transition from static components to autonomous, intelligent systems:

- *Rheological Engineering:* Balancing shear-thinning behavior for DIW with the high ionic liquid loading required for fast switching.
- *Interfacial Integrity:* Developing chemically bonded interfaces between dissimilar 3D-printed layers to prevent delamination.
- *Digital Maturation:* Moving toward fully autonomous 4D-printing, where AI-optimized inks are used to create systems that adapt their optical properties to complex environmental stimuli.

In conclusion, the synergistic integration of PILs, ionogels, and AM provides a robust roadmap for next-generation smart optoelectronics. Mastering the structure–property–processing relationships is not only a scientific necessity but also the key to realizing the 30–40% cost-reduction potential of digital manufacturing. While challenges in interfacial adhesion and rheological tuning persist, addressing industrial bottlenecks, such as printing throughput and long-term encapsulation via AI-guided optimization, will facilitate the transition from laboratory prototypes to commercially viable technologies. Ultimately, shifting toward a “design-by-function” paradigm will enable the widespread adoption of freeform, scalable, and cost-effective electrochromic systems across the architectural and automotive sectors.

## Figures and Tables

**Table 3:** Comparison of AM modalities for ECDs.

AM Technique	Materials	Resolution (µm)/ Printing Speed (mm/s) *	Key Advances	Key Limitations	ECD Examples
**DLP/SLA**	Photocurable ionogels, PIL resins	High (10–50)/ Fast (0.01–0.1) ** (whole layer curing)	High geometric fidelity	Oxygen inhibition, limited multi-material capability	Micro-patterned displays, high-res sensors [54]
**DIW**	Viscous ionogels, hydrogels, pastes	Medium (100–500)/ Slow to Medium (10–100)	Multi-material integration, wide material viscosity range	Interfacial delamination, lower resolution	Wearable FECDs, monolithic smart windows [55,61]
**Inkjet** **printing**	Low-viscosity PIL solutions, nanomaterial inks	Very High (20–50)/ Fast (100–500) (for thin films)	Precise patterning	Low layer thickness, strict viscosity constraints	Smart textiles, image-integrated windows [146,147]
**FFF**	Thermoplastic composites (PLA/IL, TPU/IL)	Low (200–400)/ Medium (10–100)	Low cost, accessibility, structural strength	Filament buckling at high IL loading, thermal degradation risk	Structural electrodes, prototyping housings [150]

* The speed and acceleration parameters determine the self-vibrations of the printer. ** Converted from vertical build rates (mm/h) to mm/s for standardization purposes.

## Data Availability

Data is contained within the article.

## Abbreviations

The following abbreviations are used in this manuscript:

AI	Artificial intelligence
AM	Additive manufacturing
CAGR	Compound annual growth rate
CE	Coloration efficiency
DAE	Diarylethene
DIW	Direct ink writing
DLP	Digital light processing
EC	Electrochromic
ECD	Electrochromic devices
ECPDLC	Electrochromic polymer-dispersed liquid crystal
EL	Electroluminescent
IL	Ionic liquids
ML	Machine learning
MOFs	Metal–organic frameworks
NIR	Near-infrared
PANI	Polyaniline
PDMS	Polydimethylsiloxane
PE	Polyethylene
PEDOT:PSS	Poly(3,4-ethylenedioxythiophene) polystyrene sulfonate
PEO	Poly(ethylene oxide)
PIL	Poly(ionic liquids)
PMMA	Polymethyl methacrylate
